# Characterization of a new strain of *Metarhizium novozealandicum* with potential to be developed as a biopesticide

**DOI:** 10.1080/21501203.2021.1935359

**Published:** 2021-06-18

**Authors:** Laura F. Villamizar, Gloria Barrera, Mark Hurst, Travis R. Glare

**Affiliations:** a AgResearch Ltd., Lincoln Research Centre, Christchurch, New Zealand; b Corporación Colombiana de Investigación Agropecuaria, AGROSAVIA,Bogotá, Colombia; c Bio-Protection Research Centre, Lincoln University, Christchurch, New Zealand

**Keywords:** *Metarhizium*, entomopathogenic fungus, porina, grass grubs, diamond back moth, microsclerotia

## Abstract

The fungal species *Metarhizium novozealandicum*, that occurs only in New Zealand and Australia has been poorly studied.  In this work, a new strain of *M. novozealandicum* isolated from a larva of *Wiseana* sp. is described based on morphology, genomic multilocus (ITS, EF-1α and β-tubulin) phylogeny, growth in different culture media and insecticidal activity. The isolate AgR-F177 was clustered in the same clade with *M. novozealandicum*. AgR-F177 colonies developed faster on Sabouraud Dextrose Agar (SDA) than on Potato Dextrose Agar (PDA) when incubated at 25°C, with no growth observed at 30°C on either media. Conidia yield on an oat-based medium in semisolid fermentation was 7.41 x 10^8^conidia/g of substrate and a higher yield of 1.68 x 10^9^conidia/g of substrate was obtained using solid fermentation on cooked rice. AgR-F177 formed microsclerotia (MS) in liquid fermentation after 7 days reaching the maximum yield of 3.3 × 10^3^ MS/mL after 10 days. AgR-F177 caused mortality in *Wiseana copularis, Costelytra giveni* and *Plutella xylostella* larvae with efficacies up to 100%, 69.2%, and 45.7%, respectively. The ease of production of AgR-F177 with different fermentation systems and its pathogenicity against different insect pests reveal its potential as a new biopesticide.

## Introduction

Insect pests are a major problem for New Zealand’s primary industries, causing millions of dollars of lost production each year (Ferguson et al. 2018). Some insect pests are endemic species that have adapted to introduced pasture plants and some horticultural crops to become significant pests (Popay 2008). One of the most significant pests is Porina, an endemic insect pest in the genus *Wiseana* (Lepidoptera: Hepialidae), found throughout New Zealand affecting most pasture species including clover and ryegrass. Seven *Wiseana* species are currently recognised in New Zealand: *W. cervinata, W. copularis, W. fuliginea, W. jocosa, W. mimica, W. signata,* and *W. umbraculata*(Richards et al. 2017). All species are univoltine. The larvae negatively affect pasture production (Ferguson et al. 2018). Another significant native pest in pasture is the New Zealand grass grub *Costelytra giveni*(Coleoptera: Scarabaeidae), formerly *C. zealandica* (Coca-Abia and Romero-Samper, 2016). Devastating outbreaks of this pest can be caused by large-scale land changes, such as implementation of irrigation schemes or conversion from forestry (Jackson et al. 2012). The control of this pest costs farmers in the dairy, sheep and beef industries between 41 USD million and 90 USD million annually in lost production (Popay 2008). There are also introduced pests as *Plutella xylostella* (Lepidoptera: Yponomeutidae) commonly known as diamond back moth (DBM), a globally destructive pest of brassicaceous crops, which has developed resistance to many conventional and novel insecticides (Perry et al. 2020).

Current strategies to control these pests relies on synthetic pesticides such as diazinon and chlorpyrifos, organophosphorus products with a mode of action based on their ability to inhibit the enzyme acetylcholinesterase (AChE), leading to the accumulation of neurotransmitters and altered signal transmission in chemical synapses (Pham and Bui 2018). Non-discriminate organophosphate insecticide toxicity is considered a major global health problem and several molecules have been banned in different countries, with the New Zealand Environmental Protection Authority intending to be phased out diazinon by 2028 ([EPA] Environmental Protection Authority 2013).

Biological control using entomopathogenic fungi, such as *Metarhizium anisopliae* (Metchnikoff) Sorokin (1883) *sensulato* (Ascomycota, Hypocreales, Clavicipitaceae) (Latch and Kain 1983), and bacteria including *Yersinia entomophaga* (Yersiniaceae) (Hurst et al. 2020) and *Serratia entomophila* (Enterobacteriaceae) (Wright et al 2017) is an environmentally friendly and sustainable strategy to manage these pests. However, only *S. entomophila* has been developed as a commercial biopesticide (Bioshield™) available in the market for specifically managing grass grub (Zydenbos et al. 2016).

For new fungal biological control agents for insect pests to be effective, new isolates must not only be virulent, but also able to be produced easily and their persistence and environmentally safety assured, which requires specific taxonomic identification. This is particularly important for selection of new isolates of the hyphomycetous fungus *Metarhizium* spp. (Fernandes et al. 2010). *Metarhizium* spp. are ubiquitous naturally occurring soil inhabiting fungi, and some are rhizosphere colonisers, with occurrence of species attributed to various selective factors (habitat type, climatic conditions, specific associations with plants and insect hosts) (Brunner-Mendoza et al. 2019). *Metarhizium* species have the ability to directly penetrate the insect cuticle through a combination of mechanical pressure and cuticle-degrading enzymes. When attaching themselves to the body of a suitable host, conidia produce a germ tube which, through extension and growth, give rise to hyphae that penetrate into and grow within the insect, eventually leading to death (Beys-da-silva et al. 2014).

The genus *Metarhizium* is composed of several species of entomopathogenic fungi mostly characterised by producing green conidia that cover the host cadaver, which is why it has been termed the “green muscardine fungus” (Nishi and Sato 2018). This genus has been recently revised with the description of several new species and reclassifying some species from other genera. The most recent review was carried out by Mongkolsamrit et al. (2020), who revisited the genus and described 19 new species from Thailand: *M. clavatum, M. sulphureum, M. biotecense, M. fusoideum, M. culicidarum, M. nornnoi, M. megapomponiae, M. cicadae, M. niveum, M. candelabrum, M. cercopidarum, M. ellipsoideum, M. huainamdangense, M. ovoidosporum, M. eburneum, M. phuwiangense, M. purpureum, M. purpureonigrum and M. flavum*. According to this review, the genera currently comprises 51 species.

The insect host ranges vary within the genus with some species exhibiting a broad host range, such as *Metarhizium robertsii*infecting insect species from more than seven orders, as well as arachnids, while others, such as *Metarhizium acridum*, have a narrow known host range, restricted to the order Orthoptera (Brunner-Mendoza et al. 2019).

A recent reclassification of this genus placed *Metarhizium novozealandicum* (Driver & Milner) Kepler, S.A. Rehner& Humber Kepler et al. 2014, previously associated with *Metarhizium flavoviride* complex, outside this complex (2014).This New Zealand-endemic fungus has been poorly studied despite its potential as a biological control agent. To date, there are no reports that describe its morphology, physiology, mass production, insecticidal activity and much less its use as a biopesticide. In this study, we report a new strain of *M. novozealandicum*, designated AgR-F177, which was isolated from a naturally infected larva of Porina *(Wiseana*sp.) collected in Methven (Canterbury, New Zealand). The aims of this study were (1) to genetically identify the isolate AgR-F177, (2) to morphologically characterise the fungal macroscopic growth and microscopic structures, (3) to assess the feasibility of massively produce conidia and microsclerotia, and (4) to assess the pathogenicity against larvae of three insect pests. This work will provide the basis for more detailed research on *M. novozealandicum*as a potential candidate to develop fungus-based pesticide products and their large-scale application for biological control of pests in pastures in New Zealand.

## Materials and methods

### Fungal strain

The strain AgR-F177 was obtained from the fungal collection of AgResearch Limited (Lincoln, New Zealand) where it is maintained as a conidial suspension in glycerol (15%) at −80°C. The fungus was grown on Potato Dextrose Agar plates (PDA, Oxoid BO1010M) at 25°C in dark conditions for 10 days allowing the production of conidia required for inoculating the fermentation. Conidia for DNA extraction was produced using semi-solid fermentation on oat-based medium according to the methodology described by Mejía et al. (2020).

### DNA extraction and phylogenetic analysis

Genomic DNA was extracted directly from frozen conidia after being ground with liquid N_2_. DNA extraction was conducted using the Fungal DNA MiniPrep system (Zymo Research) according to the manufacturer’s instructions and recommendations for the extraction of fungal DNA.

For phylogenetic analysis, partial gene sequences were obtained for three regions; the internal transcribed spacer region of nuclear ribosomal DNA (ITS), elongation factor 1-alpha (EF-1α) and beta tubulin (β-tubulin). ITS was amplified using the primers, ITS 1 (5ʹ-TCGGTAGGTGAACCTGCGG-3ʹ) and ITS 4 (5ʹ-TCCTCCGCTTATTGATATGC-3ʹ) (White et al., 1990), β-tubulin was amplified using degenerate primers betatubF (5-TGGGCYAARGGYCACTACACYGA-3) and betatubR (5´-TCAGTGAACTCCATCTCRTCCAT-3´) (Tartar et al., 2002) and EF-1α was amplified using the primers EF1T (5´-ATGGGTAAGGARGACAAGAC) and EF2T (5´-GGAAGTACCAGTGATCATGTT-3´) (Rehner and Buckley 2005). The PCR products were cloned in TOPO-TA vector (Invitrogen, Carlsbad, CA, USA) and *Escherichia coli* Top 10 (chemo competent cells; Invitrogen, CA, USA), according to the manufacturer’s recommendations. The plasmid DNA isolated from Luria-Bertani cultures was sequenced in both forward and reverse directions) using universal primers (MACROGEN, Seoul, South Korea).The sequences were edited and aligned to obtain a consensus sequence that was compared to the representative sequences of different *Metarhizium* species from the database of the National Center for Biotechnology Information (NCBI) (Table 1).

**Table 1.** Sequences used in phylogenetic analysis

Specie and strain code	Analysed region	GenBank accession numbers
*Metarhizium flavoviride* var. *novazealandicum* strain FI 1125 (DAT F368)	ITS	AF139853.1
*Metarhizium flavoviride* var. *novazealandicum* strain FI 1124 (DAT F220)	ITS	AF139852.1
*Metarhizium flavoviride* var. *novazealandicum* strain FI 698 (F10)	ITS	AF139851.1
*Metarhizium flavoviride* var. *novazealandicum* isolate F530	ITS	DQ385622.1
*Metarhizium novozealandicum* isolate AGF99	EF-1α – β-tubulin	MK054172.1 -MK054127.1
*Metarhizium novozealandicum* isolate AGF628	EF-1α – β-tubulin	MK054165.1 – MK054120.1
*Metarhizium novozealandicum* isolate AGF401	EF-1α – β-tubulin	MK054157.1 – MK054112.1
*Metarhizium novozealandicum* isolate AGF387	EF-1α – β-tubulin	MK054156.1 – MK054111.1
*Metarhizium novozealandicum* isolate AGF264	EF-1α – β-tubulin	MK054154.1 – MK054109.1
*Metarhizium novozealandicum* isolate AGF229	EF-1α – β-tubulin	MK054151.1 – MK054106.1
*Metarhizium novozealandicum* isolate AGF178	EF-1α – β-tubulin	MK054150.1 – MK054105.1
*Metarhizium novozealandicum* isolate AGF148	EF-1α – β-tubulin	MK054145.1 – MK054100.1
*Metarhizium novozealandicum* isolate AGF133	EF-1α – β-tubulin	MK054141.1 – MK054096.1
*Metarhizium novozealandicum* isolate AGF10	EF-1α – β-tubulin	MK054137.1 – MK054092.1
*Metarhizium novozealandicum* isolate NC1038	EF-1α – β-tubulin	MK054136.1 – MK054091.1
*Metarhizium novozealandicum* isolate NC1035	EF-1α – β-tubulin	MK054134.1 – MK054089.1
*Metarhizium novozealandicum* isolate BCF30	EF-1α – β-tubulin	MK054133.1 – MK054088.1
*Metarhizium anisopliae*	ITS – EF-1α – β-tubulin	PRJNA530366
*Metarhizium acridum* CQMa 102	ITS – EF-1α – β-tubulin	PRJNA245139
*Metarhizium rileyi* RCEF 4871	ITS – EF-1α – β-tubulin	PRJNA72739
*Metarhizium majus* ARSEF297	ITS – EF-1α – β-tubulin	PRJNA302308
*Metarhizium brunneum* ARSEF 3297	ITS – EF-1α – β-tubulin	PRJNA608152
*Metarhizium guizhouense* ARSEF 977	ITS – EF-1α – β-tubulin	PRJNA184755
*Metarhizium robertsii* ARSEF23	ITS – EF-1α – β-tubulin	PRJNA245140
*Metarhizium album* ARSEF1941	ITS – EF-1α – β-tubulin	PRJNA72731
*Beauveria bassiana* ARSEF 2860	ITS	PRJNA225503

The phylogenetic trees were inferred by using the Neighbour-Joining method (Saitou and Nei 1987) with bootstrap consensus tree inferred from 1000 replicates. The evolutionary distances were computed using the Kimura 2-parameter method (Kimura 1980). There was a total of 482 positions in the final dataset for ITS and 1217 positions in the final dataset for concatenated sequences of β-tubulin and EF-1α. Analyses were conducted in MEGA X software (Kumar et al. 2018).

### Morphological characterisation

*Colony diametral growth vs pH*: Conidia from a 10-day-old culture on PDA were dislodge and suspended in 0.5% Tween 80 solution. Conidia concentration was determined using an improved Neubauer chamber and conidial suspensions were adjusted to 1 × 10^6^ conidia/mL. For determination of diametral growth, 2 µL drops of the conidia suspension were placed in the centre of Petri dishes with Sabouraud Agar (SDA, OxoidCM0041) and Potato Dextrose Agar (PDA, Oxoid BO1010M) adjusted to pH 5, 7 and 9 and incubated at 25 ± 1°C and 30 ± 1°C for 10 days. The diameter (D) of the fungal colony was measured with a calliper. Five replicate plates were used per treatment.

*Conidiophores and conidia morphology*: The micromorphology was evaluated according to the microculture technique described by Rivalier and Seydel (1932) with some modifications. Briefly, water-agar medium was solidified in Petri plates, and blocks (1 cm x 1 cm) were cut out with a sterile blade and placed on sterile microscope slides. Blocks were thenlaterally inoculated with conidia using a needle, covered with a cover slide and placed into Petri dishes containing water-agar to work as a humidified chamber. Plates were incubated at 25°C in for 72 hours and slides were observed at 400X magnification using an Olympus BX-50 microscope. Photos were taken using an Olympus DP72 digital camera and thirty conidia and phialides were measured using Olympus cellSensesoftware (v.1.14) (Olympus, Center Valley, PA).

### Conidia production in semisolid and solid fermentation

Conidia production in semisolid fermentation (SSF) was carried out following the methodology described by Mejía et al. (2020) with modifications. Oats were mixed with distilled water (10% w/w) and homogenised for 1 min with a kitchen blender. The substrate (200 g) was then poured into aluminium foil trays (18 cm × 11.8 cm × 4 cm), which were sterilised at 120°C for 15 min and cooled to room temperature before inoculation. The surface of the semi-solid substrates of three trays was inoculated by spraying 5 mL of conidialsuspension (1 × 10^6^ conidia/mL) per tray, which were then covered with a translucentplastic film. Solid fermentation (SF) was carried out in three 500 g capacity bags (High Density Polyethylene) containing 100 g of long grain white rice and 80 mL of water. Bags were sterilised by autoclaving at 121°C, 15 psi for 15 mins and inoculated with 5 ml of 10^6^ conidia/mL.

Inoculated trays and bags were incubated in a growing room with 24 h artificial light (Philips 24 W/840Master TL5 HO tube cool white) at 23 ± 2°C for 14 d. One disc (10 mm in diameter) of colonised substrate was removed from differentareas of each tray at 4, 6, 8, 10, 12 and 14 d of incubation to estimate conidia yield. The disk from each tray was weighed, suspended in 9 mL of Tween® 80 solution (0.5% v/v) and homogenised for conidia counting in a Neubauer chamber. At the same sampling times, 1 g of sporulated rice was removed from each bag and washed in 9 mL of Tween® 80 solution (0.5% v/v) for conidia counting using a Neubauer chamber.

## Microsclerotia (MS) formation in liquid culture

Liquid culturing was conducted in non-baffled 250 mL conical flasks containing 100 mL of MS culture media (Mascarin et al. 2014) with a carbon concentration of 16 g/L and a C/N ratio of 50:1. The liquid medium had the following composition (per litre): glucose 36 g; yeast extract 3.64 g; KH_2_PO_4_, 4.0 g; CaCl_2_.2H2O, 0.8 g; MgSO_4_.7H_2_O, 0.6 g; FeSO_4_.7H_2_O, 0.1 g; MnSO_4_.H_2_O, 0.016 g; ZnSO_4_.7H_2_O, 0.014 g.

Flasks were inoculated with 1 mL of a 10^6^ conidia/mL spore suspension. Cultures were grown at 25°C and 300 rpm on a benchtop rotary shaker (Infors HT Ecotron). Each flask was vigorously hand-shaken once daily for the first five days to remove any mycelia growth from the sides of the flasks, thereafter any mycelial rings that formed on the side of the flasks were aseptically removed with a sterile loop at subsequent sampling times. On days 4, 7, and 10, 1 mL samples of whole culture broth were aseptically collected. To check for MS formation, 100 μL of culture broth was placed on a glass side and overlaid with a 22 × 50 cm glass coverslip, and the number of MS were counted across the entire coverslip area. Blastospores concentration was also determined by counting with a haemocytometer. Three replicate flasks for each isolate and media treatment were used, and the experiment was repeated twice. Microscopic observations were made with an Olympus BX-50 microscope and photos captured using an Olympus DP72 digital camera.

MS containing biomass was harvested on day 10 by adding 5 g of diatomaceous earth Celite® 281 to each 100 mL of culture broth, and then vacuum-filtering in a Buchner funnel through Whatman No. 1 filter paper to remove spent media (Kobori et al. 2015). The resulting filtered cake was air-dried overnight under laminar flow at 22°C. MS viability was evaluated using the method described by Villamizar et al. (2018). MS granules (250 mg) were resuspended in 1 mL of 0.05% Tween® 80 (Sigma) and 100 μL samples inoculated onto water agar plates (1.5% agar w/v) using spread plate techniques and incubated at 25°C. MS viability was measured by determining the presence of hyphal growth after 48 h incubation (germination). For each water agar plate, all MS were observed using a stereomicroscope (Olympus SZX12). The total number of MS and the number of MS displaying hyphal growth were recorded and germination rate was then calculated as a percentage ratio.

Some MS were recovered from the agar plates after 24 and 48 hours incubation and observed by Electron Microscopy (JEOL 1400 plus). In this instance, MS were directly fixed with glutaraldehyde 2.5% (pH 7.4) and dehydrated with ethanol in ascendant concentrations. The samples were sputtered with colloidal gold and observed in an electron microscopy (JEOL JSM 7000 F).

## Pathogenicity assays

*Against Porina*: The bioassay was set up in 15mL specimen containers (45 mm in diameter × 60 mm in height) with a single small hole in each lid to allow ventilation. A 50:50 mix of potting mix and sieved soil was used to fill the tubes up to the 60 mL mark. One sporulated culture of AgR-F177 on PDA was used to prepare the fungus suspension in 0.01% Triton X-100, which was then adjusted to 1 × 10^8^ conidia/mL. Samples of 1 ml were applied across the top of the soil surface in each container (14 containers/treatments). Control treatment was applied with 1 mL of 0.01% Triton X-100 per container. *W. copularis* larvae (6^th^ instar porina larva of approximately 25–35 mm in length) collected in the North Island and held at 4°C until use were sorted according to size, and one larva was added to each treated container. Each container then received a single clover leaflet and, after being capped, they were placed into an incubator at 15°C with a layer of dry paper sheets over the top of the tubes to prevent the soil drying out too quickly. On day 3, tubes were checked to ensure the larvae had burrowed and were feeding. Clover (*Trifolium repens* L.) leaves were added each week to each container. Larvae were assessed for feeding and mortality at seven days intervals. Tap water was added to soil surface where there were signs of the soil drying out. Ryegrass and clover were provided at each assessment time.

*Against Grass Grub*: The bioassay was set up in 15 mL test tubes (25 mm in diameter × 80 mm in height) containing 10 g of dry soil (48 h oven dry at 80°C). One sporulated culture of AgR-F177 on PDA was used to prepare the fungus suspension in 0.01% Triton X-100, which was then adjusted to 1 × 10^8^ conidia/mL. Samples of 2 ml were applied across the top of the soil surface in each container (30 containers/treatments). Control treatment was applied with 2 mL of 0.01% Triton X-100 per container. Healthy third-instar larvae collected in the South Island and quarantine for 15 days at 15°C were individually added to each treated container. Each container then received a 3 mm cube of fresh carrot and, after being capped, were distributed into plastic containers (10 tubes/container and 3 containers/treatment) and incubated at 22°C in the dark. Tubes were checked weekly and carrot cubes were added each week to each container. Mortality was assessed at seven days intervals for three weeks.

*Against Diamond Back Moth*: The bioassay was set up in 22 mL cups (25 mm in diameter × 20 mm in height). One sporulated culture of AgR-F177 on PDA was used to prepare 10 mL of fungus suspension in 0.01% Triton X-100 adjusted to 1 × 10^8^ conidia/mL. The fungal suspension was poured in a Petri dish, and 4 discs (20 mm diameter) of cabbage leaves were dipped into the suspension. After 30 seconds, all discs were turned upside-down and incubated for 30 additional seconds and then were transferred to a dry Petri dish to allow them to dry. Control treatment consisted of 4 cabbage discs treated with 0.01% Triton X-100. Each treated and dry disc was placed in one 14 mL cup and infested with 10 healthy second-instar larvae of *P. xylostella*(obtained from the colony maintained at Lincoln University, Canterbury, New Zealand), for a total of 40 larvae/treatments. Containers were capped and incubated at 20°C in the dark for 7 days, were larvae mortality was assessed.

For all the bioassays, mortality in the treatments was corrected with the mortality in the control by using the Schneider-Orelli formula, calculating efficacy (Zar 1999).

$$Efficacy\left(\%\right)=\frac{\left(A-C\right)}{\left(100-C\right)}x100$$

where,

A = Mortality in the treatment

C = Mortality in the control treatment

## Data analysis

Data from diametral growth and conidia yield were checked for normality and homogeneity of variance using Shapiro–Wilk and Barlett tests, respectively. The diametral growth rate of each sample for each time period was calculated by subtracting the measured diameter value of the former time point from the value of the latter time point. Then, since the growth rate measurements were taken repeatedly from the same set of samples over the whole experimental period, the growth rates were analysed using a linear mixed model (LMM). The LMM consisted of full factorial combination of three factors: Medium (two levels – PDA or SDA), pH (three levels – 5, 7, or 9) and Period (four levels – 2-4 days, 4–6 days, 6–8 days, or 8–10 days). The LMM analysis was carried out with statistical software SAS version 9.4 (SAS Inst., Cary, NC.). Mean values for all the other experiments were compared by one-way ANOVA and Tukey test (95%) using Statistix 8.1 (Analytical Software, Tallahassee, FL, USA).

## Results

### Phylogenetic analysis

**Figure 1.**
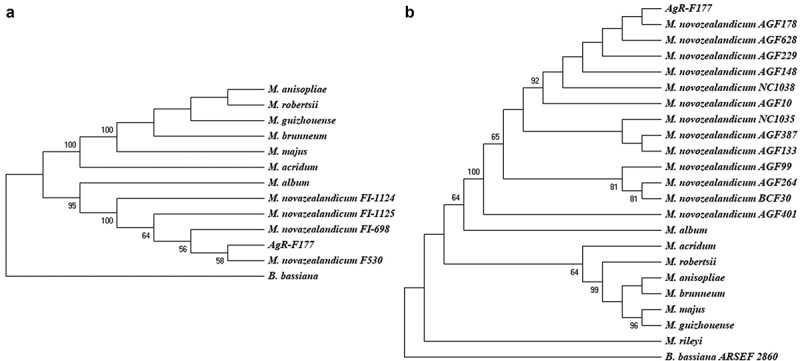
Phylogenetic trees constructed with kimura 2-parameter distance and Neighbour-Joining method. A. Tree constructed with ITS sequences, B. Tree constructed with concatenated sequences ofEF-1α and β-tubulin.bootstrap values higher than 50 are shown above of each branch

For the phylogenetic analysis, two trees were constructed, one based on ITS and the other based on concatenated sequences of EF-1α and β-tubulin. For the analysis, sequences of different species of *Metarhizium* were used and a sequence from *Beauveria bassiana*was used as outgroup. In the tree built with the ITS sequences, *Metarhizium* species separated in two groups, one in which *M. novozealandicum* and *M. album* are located and a second one grouping the other species (Figure 1A).

Based on the ITS sequence, the strain AgR-F177 is grouped with 95% bootstrap value with all the strains reported by Driver et al. (2000) and Reay et al. (2007) as *M. novozealandicum* (Figure 1A), that have been deposited in the Genbank and correspond to Australian isolates obtained from soil samples (FI-1124 and FI-1125) and New Zealand isolates obtained from insect specimens (FI-698 and F530).

The analysis based on the concatenated sequences of EF-1α and β-tubulin showed similar topology, with AgR-F177 strain grouped with all the other *M. novozealandicum*in a well-supported clade (100% bootstrap value), while the other *Metarhizium*species were included in another group. The isolates AGF148, AGF178, AGF229, AGF628, AGF10, AGF401, AGF387, AGF133, AGF99 and AGF264, NC1035 and NC1038 of *M. novozealandicum* used for the comparison in the present work were isolated in New Zealand and their sequences were deposited by Cummings and Glare in the Genbank in 2018.

The strain NC1035 presented the highest genetic distance (0.008) to AgR-F177 (Table S1) within all *M. novozealandicum*isolates. Genetic distance among different species were higher than 0.100.

### Morphological characterisation

#### Colony diametral growth vs pH

**Figure 2.**
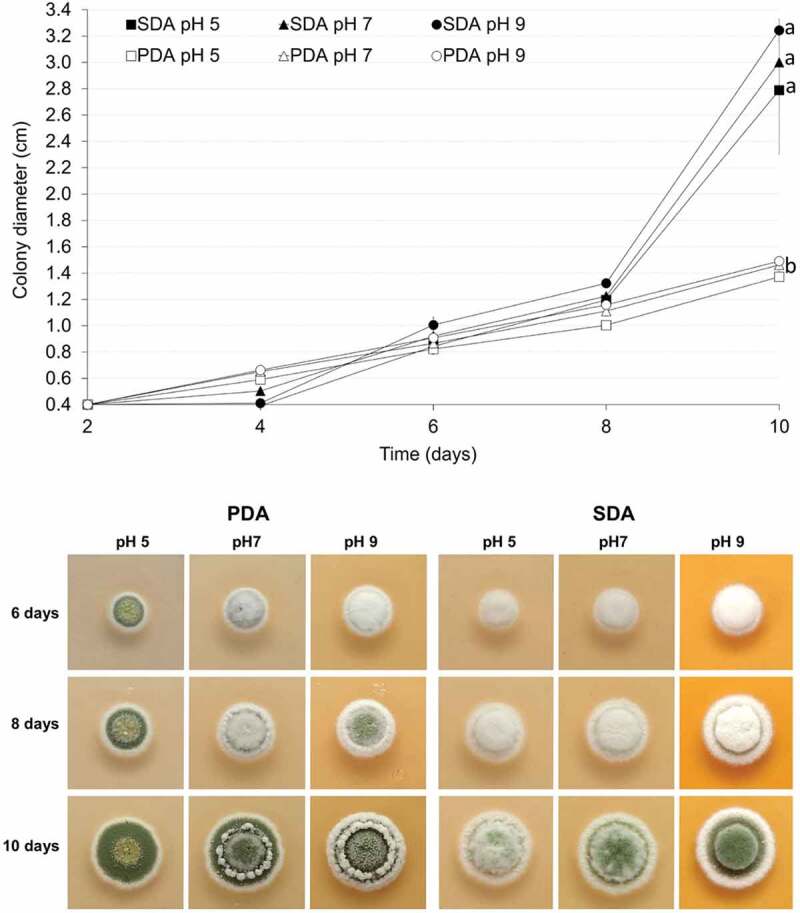
Comparative analysis of colony diametral growth on SDA and PDA at different pH at 25°C

All cultures of *M. novozealandicum* AgR-F177 grew and sporulated at 25°C but no growth was observed at 30°C for all treatments. The size of colonies increased with age (Figure 2). Final diameters after 10 days incubation at 25°C were between 1.3 and 1.5 mm for colonies grown on PDA and between 2.7 and 3.3 mm for colonies grown on SDA (Figure 2).

Colony diameter increased similarly in both agars until day 8, but for most of the pH ranges and time periods, the growth rate was significantly higher (p < 0.05) on SDA in comparison with PDA. For the interval from day 6 to 8, the diametral growth on SDA was significantly faster than that presented from day 2 to 8 on both agars and from day 6 to day 8 onPDA at the three pH values. During the same time interval (day 8 to day 10), the diametral growth on SDA was significantly faster at pH 9 and 7 that that obtained at pH 5 (p = 0.049 for 5 vs 7 and p < 0.001 for 5 vs 9).

Two basic sporulation colours were observed (Figure 2): greyish green (SDA) and olive green (PDA), with the second colour further differentiated into lighter or duller shades. Sporulation colour was observed to be a stable character within the five replicates (plates). Sporulation in all cultures occurred at first on the surface mycelium, which was low and formed a dense layer of growth immediately on the surface of the medium. In cultures where the overlaying aerial mycelium was well developed, the sporulation then continued in and on the aerial mycelium.

On PDA, colonies were circular with entire margin. The mycelium was flat at pH 5 and elevated at pH 7 and 9. Sporulation started sooner with the colonies covered with powdery and olive-green sporulationfrom day 6 at pH 5. Sparse sporulation in the aerial mycelium was observed at day 6 and 8 at either pH 7 and 9 and colonies were completely cover with olive-green conidia at day 10. However, a ring of elevated white mycelium grown on the top of the sporulated colonies being more abundant and pronounced at pH 9 (Figure 2). On SDA, colonies were circular, umbonate and with cottony texture due to the elevated mycelium. Greyish green sporulation was observed on day 10 at three evaluated pH (Figure 2). However, sporulation was diffuse when the agar was adjusted to pH 5 and more abundant when the pH was increased to 7 and 9.

**Figure 3.**
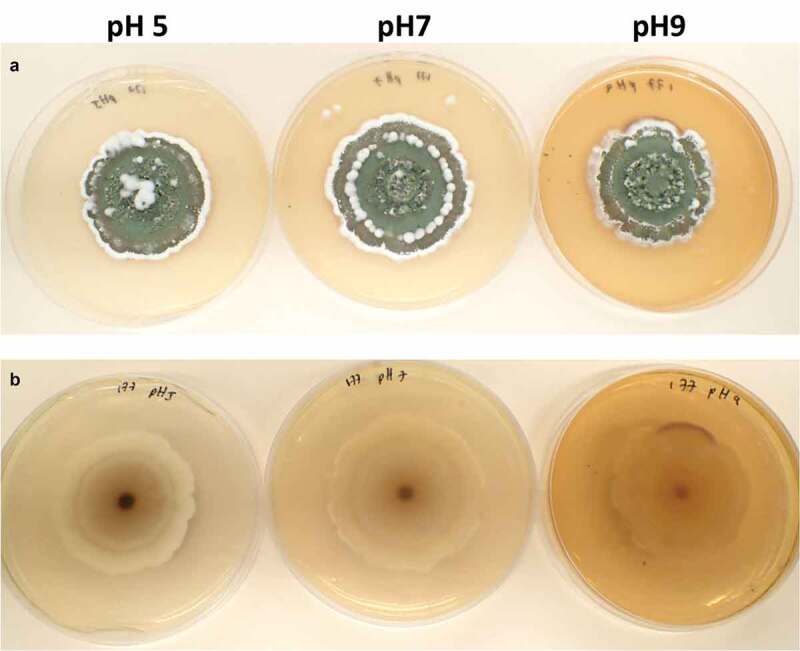
Twenty days-old colonies on PDA adjusted at different pH. A. obverse B. reverse

Twenty days old AgR-F177 colonies on PDA were circular with undulate margin and powdery texture due to the abundant sporulation which was olive green in the central part of the colony and brownish green close to the edges. While observing through the reverse side of the plate the colony colour varied from whitish yellow, through shades of yellow to shades of purple (Figure 3).

#### Conidiophores and conidia morphology

**Figure 4.**
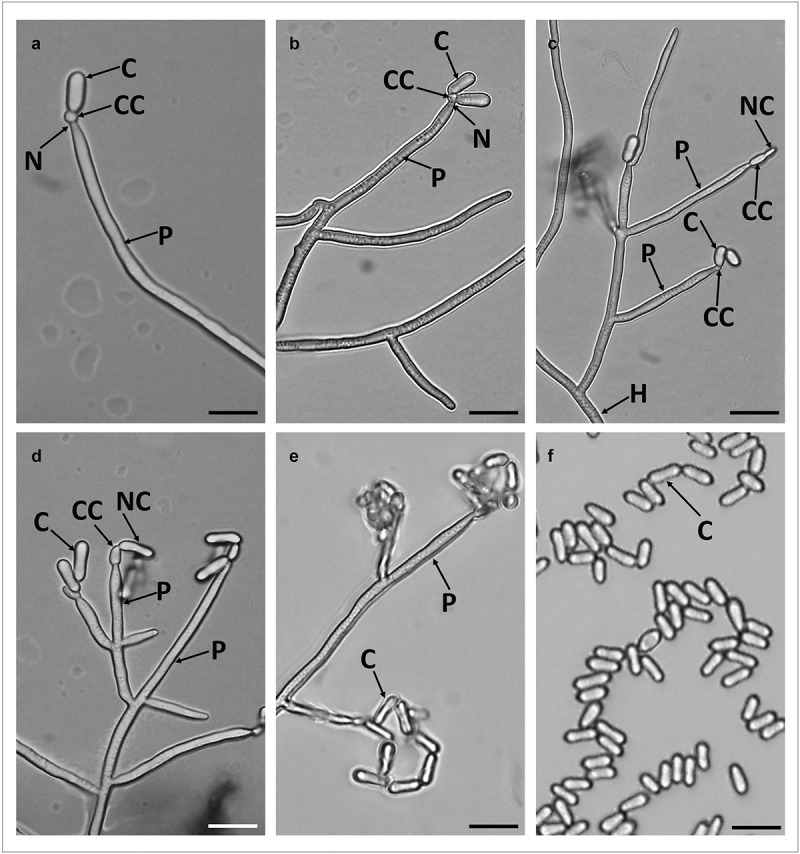
Micrograph of branched conidiophores and conidia formed by *M. novozealandicum* AgR-F177 on water agar. Conidium (C), Phialide (P), Neck (N), Conidiogenous cell (CC), Hyphae (H), Newly formed budding conidium (NC). Scale bars: 10 µm

Loosely branched conidiophores arise from hyphae with each branch bearing 2–6 phialides. Phialides are long and cylindrical (24.54 ± 7.11 µm length x 1.51 ± 0.09 µm width), forming directly on the conidiophores and have a wide and short neck. The conidiogenous cells are spherical changing to ellipsoid during conidiogenesis (Figure 4C). Conidia are one-celled, smooth walled and cylindrical with rounded apex and base. In most of the conidia, side walls were straight, but some also had slightly incurving side walls so that both ends of the conidium appeared slightly swollen (Figure 4D and 4F). The overall conidia length was 5.70 ± 0.58 µm and width in 2.13 ± 0.45 µm (Figure 4F,4B and 4C).

### Conidia production in semi-solid and solid fermentation

To economically produce conidia, rice and oat substrates were assessed. Both substrates (rice and oat) were able to support *M. novozealandicum* AgR-F177 growth and sporulation. The average conidial concentrations of three replicates are presented in Table 2.

**Table 2.** Mean conidia yield and standard deviation (SD) of *M. novozealandicum* AgR-F177 produced by SSF and SF on oat and rice respectively. Values with different letter are significantly different according Tukey test (95%)

Fermentation/Substrate	Days	Yield (conidia/g)	SD (conidia/g)	Significance
**SSF/Oat**	0	2.50 x 10^4^	-	-
	7	1.09 x 10^8^	7.07 x 10^7^	***b***
	10	4.11 x 10^8^	1.09 x 10^8^	***a***
	13	4.93 x 10^8^	1.29 x 10^8^	***a***
	16	7.09 x 10^8^	1.41 x 10^8^	***a***
	20	7.41 x 10^8^	1.30 x 10^8^	***a***
**SF/Rice**	0	2.50 x 10^4^	-	-
	7	3.19 x 10^8^	1.97 x 10^7^	***d***
	10	4.07 x 10^8^	2.28 x 10^7^	***cd***
	13	5.18 x 10^8^	6.24 x 10^7^	***c***
	16	8.37 x 10^8^	1.62 x 10^8^	***b***
	20	1.68 x 10^9^	7.74 x 10^7^	***a***

AgR-F177 rapidly colonised the surface of the semi-solid oat-based medium, which was completely covered with mycelia after 4 days incubation. The mycelia surface slowly changed from white to olive green from day 5, suggesting the beginning of conidia production. Conidia yield significantly increased (F = 15.3; df = 14; p = 0.0003) from day 7 to day 10 when concentration reached 4.11 × 10^8^ conidia/g (Table 2). Final yield at day 20 (7.41 x 10^8^ conidia/g) was almost two fold the yield at day 10, but significant differences were not detected between results obtained at both times.

On rice, the surface turned reddish or purple on day 3, without exhibiting an evident mycelial growth. On day 5, olive green areas started to appear, representing sporulation, and all the grains were completely green on day 7. The colour intensified and the substrate turned dusty with longer fermentation times. Conidia yield significantly increased (F = 99.2; df = 14; p < 0.0001) during the 20 days of fermentation reaching the maximum value on day 20 (1.68 x 10^9^ conidia/g).

## Microsclerotia (MS) formation in liquid culture

**Figure 5.**
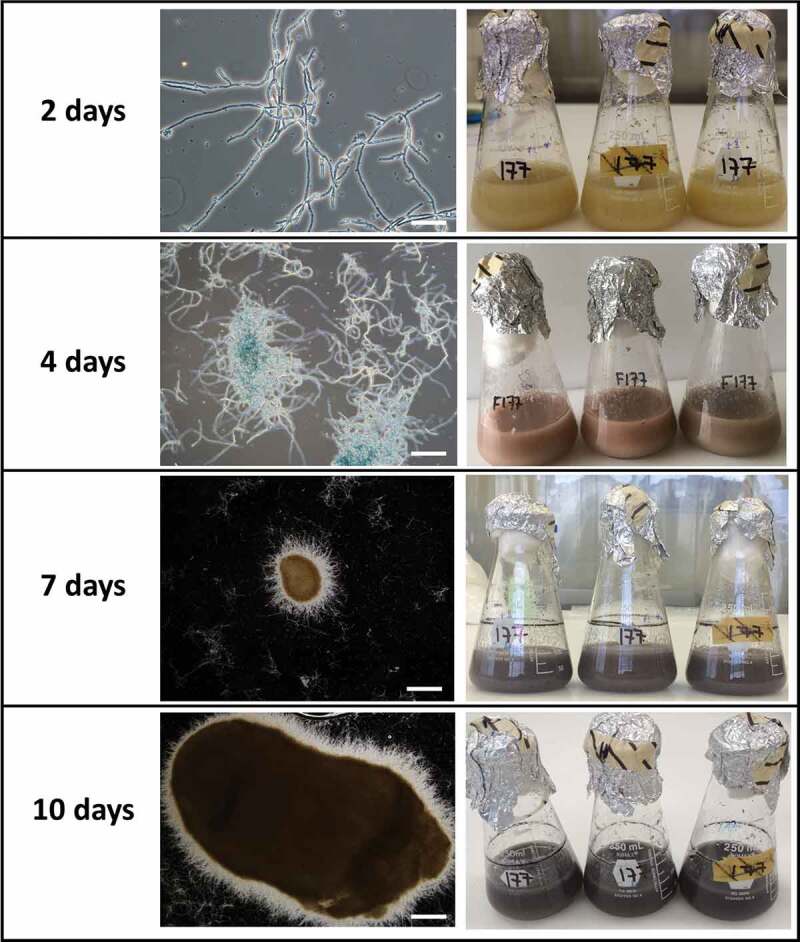
Development of microsclerotia in liquid fermentation. Scale bars: 100 µm

Germinated and non-germinated conidia, blastospores and free hyphae were observed in 2-day-old cultures (Figure 5). By day 4, the colour of the medium changed to reddish and a high concentration of blastosporeswas observed as well asincipient hyphal aggregatesthat suggested the beginning of MS formation. By day 5 of fermentation, the liquid medium started to turn light purple, then to dark purple by day 7 (Figure 5). By day 7, MS became more well-defined, compact and melanised. These structures increased in size and melanisation after day 7, also getting more compact and with shorter hyphal extensions emanating from their surface (Figure 5). The majority of MS were smaller than 400 µm (73%) and none was larger than 800 µm at day 7, while 68% of MS were larger than 400 µm at day 10 including 11% larger than 800 µm (sclerotia). At day 10, blastospores were still present (2.7 x 10^6^blastospores/mL) and MS yield was 3.3 × 10^3^ MS/mL.

**Figure 6.**
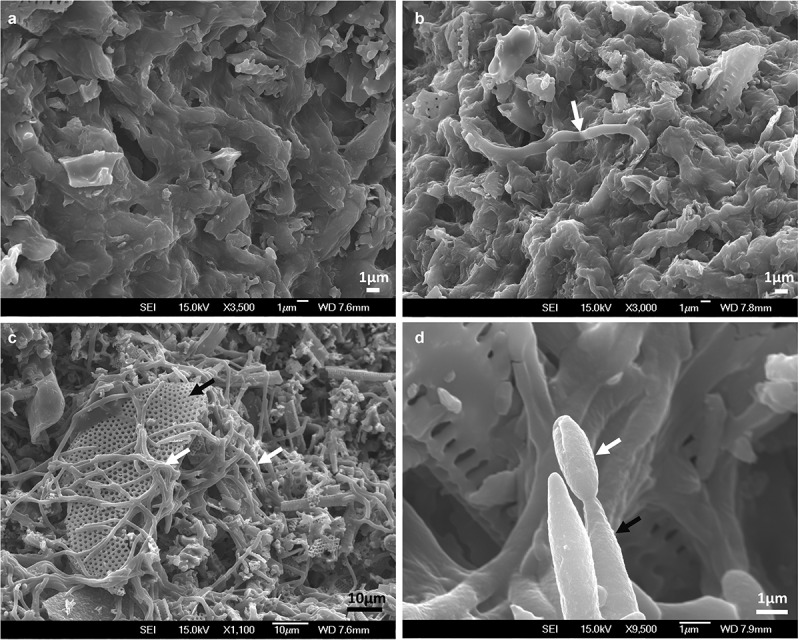
Scanning electron micrographs of MS produced by *M. novozealandicum* AgR-F177 after 24 h (A, B) and 48 h (C, D) post incubation on water agar plates. A. MS surface without hyphae growth. B. Hyphal bodies emerging from MS (white arrow). C. Mycelia (white arrow) development on MS surface where residues of diatomaceous earth (black arrow) are trapped by the fungal growth. D. Phialide (black arrow) and conidium (white arrow)

MS were harvested using diatomaceous earth to retain them during filtering. The diatomaceous earth cake formed after filtration was light purple and MS appeared as dark and compact dots. No germination was detected under the light microscope after 24 h of being inoculated on agar plates but a few hyphae were observed growing on the surface by SEM (Figure 6). After rehydration and incubation for 48 h, 100% of MS presented myceliogenic germination, with abundant hyphal growth covering the surface. Subsequently, aerial conidia were produced on hyphal extensions and on the surface of MS granules as noted by their light greenish colouration.

## Pathogenicity assay

Mortality of Porina (*W. copularis*), grass grub (*C. giveni*) and DBM (*P. xylostella*) larvae are presented in Table 3. All cadavers of Porina larvae exposed to the fungus exhibited olive green sporulation (Table 3). For grass grub and DBM larvae, fungal infection symptoms were also evidenced but only in some cadavers. where mycelia growth and green sporulation was observed (Table 3).

**Table 3.** Effect of *M. novozealandicum* AgR-F177 on *W. copularis, C. giveni* and *P. xilostella* larvae

Host	Days after inoculation	Control Mortality (%)	Treatment Mortality (%)	Efficacy (%)	Sporulated cadaver
*W. copularis / Porina*	35	35.7	100.0	100.0	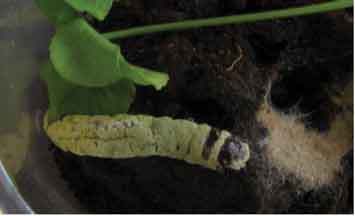
*C. giveni /* Grass grub	21	13.3	73.3	69.2	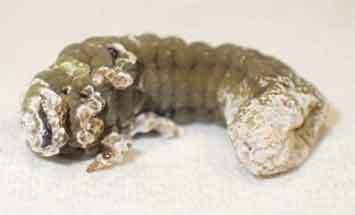
*P. xylostella /* DBM	7	12.5	52.5	45.7	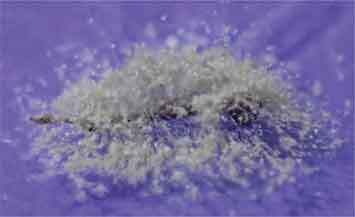

## Discussion

*Metarhizium* species are widespread in nature and have long been recognised for their biological control potential of a broad range of insects and ticks (Schrank and Vainstein 2010). *Metarhizium*conidiophores are variously branched, occasionally simple, with apices of branches bearing one to several phialides (Rombach et al. 1987). Conidia are typically green, and size and colour are used as two of the most important morphological identifying characteristics in the taxonomy of *Metarhizium*species (Chen et al. 2019). However morphological characteristics alone are now insufficient to identify all species.

Classification of fungi is very dynamic and changes frequently due new isolates being found, and more detailed genetic comparisons performed. For this reason, the use of markers with an acceptable variability that allows differentiating new species is required. The strains initially assigned to species by using the ITS sequences have been later reclassified by using other markers such as β-tubulin and EF-1α. For example, new useful molecular strategies to differentiate between closely related species of *Metarhizium*were recently described (Mayerhofer et al. 2019). In the case of *M. novozealandicum*, only ITS sequences were deposited in the Genbank for the first reported strains, however for those recently reported, the sequences of β-tubulin and EF-1α have also been included, which is why we built two separate trees for our analysis of the strain AgR-F177. All gene regions consistently grouped AgR-F177 with the other strains of *M. novozealandicum*.

The morphological and physiological characteristics of *M. novozealandicum*have been poorly described. Within the limited information available for this fungus, previous studies have mentioned that it is an endemic species from Australia and New Zealand that have been isolated from larva of *Hepialidae*spp. (Lepidoptera) and specimens of pinhole borers, *Platypus* spp.(Coleoptera), as well as from soil samples (Reay et al. 2007; Mongkolsamrit et al. 2020). Conidia have been described as cylindrical to ellipsoidal, often waisted with 5.0–7.5 µm length and 2–3 µm width (Mongkolsamrit et al. 2020), characteristics similar to those described herein for the strain AgR-F177 which had conidia 5.1 to 6.3 µm length and 1.7 to 2.6 µm width. Sporulated colonies were green, which differs from the white to pale yellow colony described by Mongkolsamrit et al. (2020), but as shown in the current study colony colour can vary with media pH and medium composition.

Using SDA and PDA, *M. novozealandicum* AgR-F177 was unable to grow at 30°C but rapidly developed mycelia and conidia at 25°C, suggesting that this strain does not tolerate hot environments. These results differ from those reported by Driver et al. (2000), who characterised two isolates of *M. novozealandicum*(one from Australia and one from New Zealand) that were able to grow over a wide range of temperatures from 10°C to 30°C. However, the colony diameters at 30°C were smaller than those obtained when incubated at lower temperatures and the strains were classified as cold-active because grew well at low temperatures (<10°C).

At 25°C, AgR-F177colonies grew at asimilar rate on SDA as PDA during the first eight days of incubation, then colonies on SDA grew faster than on PDA. SDA is composed by dextrose (40 g/L) and peptone (10 g/L) while PDA does not contain a nitrogen source (dextrose 20 g/L and potato extract 4 g/L). The type and concentration of carbon and nitrogen sources as well as C/N ratio play important roles in fungal growth and sporulation (Gao et al. 2007). In this context, the faster growth on SDA could be attributed to the higher content of nitrogen sources from the proteins contained in the peptone, considering that the fungal ability to utilise nitrate, ammonium, or organic nitrogen sources determines the extent of vegetative growth and, consequently, the reproduction capacity of the fungus (Ajdari et al. 2011). *M. novozealandicum* AgR-F177 was able to grow in a wide range of pH from 5 to 9 with faster diametral growth under alkaline conditions, possibly as a response to the alterations in the electrochemical gradient and its maintenance induced by the alkalinity (Markina-Iñarrairaegui et al. 2020). The ability of AgR-F177 to grow under an alkaline environment could suggest the presence and expression of the pH-responsive transcription factor PacC/Rim101, which governs the adaptation to environmental pH, the development and the secondary metabolism of many fungi (Wu et al. 2016).

Sporulation of AgR-F177 on PDA started faster at pH 5 than in more alkaline media, a treatment that also presented less elevated mycelia, which is consistent with the optimal pH (5–6) for sporulation reported for several others entomopathogens such as *Lecanicillium lecanii* (Vu et al., 2008), *Beauveria bassiana* (Mishra and Malik, 2013), *Metarhizium rileyi* (= *Nomuraea rileyi*) (García and Del Pozo 1999).

In general, growth of entomopathogens is optimal over a broad range of pH, which implies that these fungi may regulate cytosolic pH more effectively than many other species (Hallsworth and Magan1996). The pH response observed for AgR-F177 could be compared with other pH studies on several *Metarhizium*species conducted on solid medium. The results obtained in our study were similar to those observed for *M. rileyi* by Aguirre et al. (2009), who reported a wide range of pH for growth between 4 to 9, with maximum radial growth rate under alkaline conditions at pH 8. Similarly, strains of *M. anisopliae*are also able to grow and survive under a wide range of pH, reported as between 5 to 8.5 (Milner 1989) and 2.2 to 10.5 (JE and Magan 1996).

Rice and oats supported the growth and sporulation of *M. novozealandicum* AgR-F177 but the final yield on rice (at 20 days) was 2.27-fold more conidia than produced per gram on the oat-based medium. The higher yield per gram of substrate obtained using SF on rice in comparison with SSF on oats could be related to the larger surface area available for the fungus to grow on the grains. This is supported by the continuing increase in conidia production during the 20 days of fermentation, while only a non-significant increase was observed after day 10 using SSF. The relationship between conidia yield and available surface area has been demonstrated for conidia mass production of several fungi including *B. bassiana*(Xie et al. 2012), *M. anisopliae* (Barra-Bucarei et al. 2016) and *Trichoderma harzianum* (Mayo-Prieto et al. 2020). Rice is the most often chosen substrate to produce conidia for formulation in biopesticides, because it has easily available sources of nutrients at high concentrations, large surface areas and maintains its physical structure during sterilisation and the fermentation process (Bhanu Prakash et al. 2008). The maximum yield was obtained when AgR-F177 was grown for 20 days on rice (1.68 x 10^9^ conidia/g), resulting in the same magnitude (10^9^ conidia/g) to previous reports for other entomopathogenic fungi produced using SF on rice grains, such as *M. anisopliae, B. bassiana,* and *Beauveria brongniartii* (Nelson et al. 1996; Sahayaraj and Namasivayam 2008; Barra-Bucarei et al. 2016).

MS formation was successfully induced when AgR-F177 was cultured by liquid fermentation using the medium previously standardised for MS production in *M. anisopliae, M. robertsii* and *M. acridum* (Mascarin et al. 2014). Microsclerotia have been described as pseudoparenchymatal aggregations of hyphae that become melanised (dark) during development (Song et al. 2014). MS formation is artificially induced in entomopathogenic fungi and yield varies with the carbon concentration as well as the C:N ratio of liquid media (Jaronski and Jackson 2012). The yield obtained in the present work (10^3^ MS/mL) was similar to those reported by Rivas-Franco et al. (2020) for the isolate F99 of *M. novozealandicum*with ∼1 × 10^3^ MS/mL and those reported for other species from the same genera such as *M. anisopliae, M. robertsii* and *M. acridum*, which reached maximum yields between 7.0 × 10^3^ and 1.1 × 10^4^ MS/mL (Mascarin et al. 2014). Similar yields were also reported for the entomopathogenic fungi *B. brongniartii*and *B. bassiana*with 1.1 × 10^3^ and 3.8 x 10^3^MS/mL, respectively (Villamizar et al. 2018). Higher yields have been obtained with different fungal species when culture media composition and the fermentation conditions have been optimised, reaching values between 10^4^ and 10^5^ MS/mL (Kobori et al. 2015; Song et al. 2016). The growth conditions for MS production with *M. novozealandicum* AgR-F177 have not yet been optimised, but the culture medium and fermentation parameters evaluated in the present work could be the basis for further optimisation and scale up of this process.

MS produced by AgR-F177 were dark brown to black at day 10 of fermentation, which suggests a high level of melanisation and/or the encapsulation of produced pigments within the cellular structure. Melanisation plays a significant role in MS performance as resistance structures due to melanin capacity of mitigating the deleterious effects of UV radiation, temperature, desiccation, free radicals and metal ions (Butler and Day 1998). Melanins also have been shown to possess antimicrobial properties against antagonist organisms (Aa and MH 1986). Microsclerotia are produced by many phytopathogenic fungi as overwintering propagules that resist adverse environmental stressors (Gordee and Porter 1961; ; Griffin et al. 1978; López-Escudero et al. 2006). Several authors have demonstrated that MS from biocontrol fungi can be used in bioinsecticides (Jackson and Jaronski 2009; Song et al. 2014; Clifton et al. 2020), biofungicides (Kobori et al. 2015) or in bioherbicides (Shearer 2007) formulations.

Of note, a red colour was observed on AgR-F177 colonised rice, purple colour was observed on the reverse of the colonies grown on PDA and the MS broth changed from yellow to purple during the liquid fermentation, This colouration is indicative of the ability of AgR-F177 to produce pigmented metabolites. In this context, several metabolites from *Metarhizium* spp. have been previously identified, some of which play essential roles during fungal infection (Kikuchi et al. 2009; Kozone et al. 2009; Nishi et al. 2017). This study demonstrated that *M. novozealandicum* produces red-purple pigments under different culture conditions. These pigments could be related with insecticidal activity (virulence factors) as noted with oosporein, a red-purple pigment produced by *Beauveria* spp. which contributes with the mode of action by reducing insect haemocyte numbers and by alterations to the insect humoral immune system (Feng et al. 2015; Mc Namara et al. 2019). A similar red-purple pigment was found in the new species recently reported in Japan, *Metarhizium purpureogenum*, which also represents a unique lineage in *Metarhizium* with a weak relationship to *M. novozealandicum* (Nichi et al.^2017^). Further work is required to elucidate the identity of these red-purple pigments, their bioactivity and triggers for production, which will contribute to better understand the physiology, ecology and mode of action of *M. novozealandicum*and could lead the development of new bio-active molecules.

In bioassay assessments using a simple spore suspension *M. novozealandicum* exhibited good bioactivity towards Porina (*W.copularis*) larvae with greater than 70% lethality at 21 days and 100% mortality 35 days post challenge. In this respect the damp and cooler conditions found in a Porina burrow combined with the requirement of larvae to drag food into the burrow to feed will likely favour fungus-induced mortality. *M. novozealandicum*was also pathogenic to grass grub (*C. giveni*) reaching 69.2% efficacy at 21 days post challenge, similar to the level of control reported by Glare (1994), where 60% mortality caused by *M. anisopliae*by day 35 after inoculation was observed. Rivas-Franco et al. (2019) also reported similar mortalities, with values up to 67% when using *M. anisopliae* and up to 55% when using *M. novozealandicum*as a seed coating on maize seeds. The isolate AgR-F177 was demonstrated to be pathogenic against DBM but the efficacy only reached 45.7%, suggesting low virulence against this insect when compared with other fungal species such as *Beauveria bassiana, Metarhizium rileyi* and *Isaria sinclairii*that can reach between 80% to 100% mortality (Duarte et al. 2016). However, it is important to note that having demonstrated the pathogenicity of *M. novozealandicum*AgR-F177 against DBM, its virulence could be improved using strategies such as accelerated evolution with the host (Valero-Jiménez et al. 2017).

As mentioned before, the current strategy to control this pest relies only on synthetic pesticidesas diflubenzuron and chlorpyriphos (Pham and Bui 2018), which negatively impact in the environment (Bonifacio et al. 2017) and could be banned in the future. In this context, *M. novozealandicum*AgR-F177 represents a new alternative for a more sustainable management of insect pests. It would be worth also assessing *M. novozealandicum* in combination with the bacterial entomopathogen *Y. entomophaga* for field efficacy against Porina and Grass grub larvae (Hurst et al. 2020), considering that both biocontrol agents have different mode of action and temperature optimums, being able to be synergistic or have an additive effect when integrated in a management programme for this pest.

The high yield of conidia in SSF and Sf, the ability to produce MS in submerged culture, the pathogenicity against three important agricultural pests, coupled with known formulation and application methods for these types of entomopathogenic fungi make AgR-F177 a strong candidate for further development as a biopesticide in New Zealand.
